# The Value of High-Frequency Repetitive Transcranial Magnetic Stimulation of the Motor Cortex to Treat Central Pain Sensitization Associated With Knee Osteoarthritis

**DOI:** 10.3389/fnins.2019.00388

**Published:** 2019-04-18

**Authors:** Jean-Paul Nguyen, Véronique Dixneuf, Julien Esnaut, Alcira Suarez Moreno, Catherine Malineau, Julien Nizard, Jean-Pascal Lefaucheur

**Affiliations:** ^1^Unité de Stimulation Magnétique, Centre d’Evaluation et de Traitement de la Douleur (CETD), Clinique Bretéché, Groupe ELSAN, Nantes, France; ^2^Centre Fédératif Douleur, Soins Palliatifs et Support, Ethique Clinique et Laboratoire de Thérapeutique, Nantes, France; ^3^Unité de Neurophysiologie Clinique, CHU Henri Mondor, APHP, Faculté de Médecine, UPEC, Créteil, France

**Keywords:** alpha synuclein, Parkinson disease, lipid rafts, prion protein, amyloid precursor protein, metabotropic glutamate receptor 5, NMDA receptor

## Abstract

**Aim:**

Chronic pain associated with knee osteoarthritis may develop in connection with a maladaptive process of pain sensitization in the central nervous system. Repetitive transcranial magnetic stimulation (rTMS) has been proposed to treat various pain syndromes related to central sensitization phenomenon, but was never applied in the context of knee osteoarthritis.

**Methods:**

A 71-year-old woman presenting clinical evidence of central sensitization of pain associated with left knee osteoarthritis underwent monthly sessions of rTMS delivered at 10 Hz over the right motor cortex.

**Results:**

From the week following the third session, she began to improve on various clinical aspects, including pain. After 10 sessions (i.e., almost one year of follow-up), pain was reduced by 67%, especially regarding neuropathic components, while sleep disorders and fatigue also improved by 57–67%. The central sensitization inventory (CSI) score was reduced by 70%.

**Conclusion:**

This observation suggests that high-frequency motor cortex rTMS could be a therapeutic option to treat neuropathic pain and psychological symptoms associated with central sensitization developing in the context of chronic osteoarthritis of the knee joint.

## Introduction

Pain is the main symptom of knee osteoarthritis (Nguyen et al., 2011). It initially appears as “mechanical,” occurring when the joint is solicited, and gradually leads to joint stiffness, which can cause gait disorders and postural imbalance. The management of osteoarthritis of the lower limbs has been the subject of regularly updated recommendations, including those of EULAR in Fernandes et al. (2013). Weight loss in case of overweight and physical therapies can improve pain and peri-articular stiffness, but they are often insufficiently effective. Since pain is also related to inflammation of the joint and surrounding soft tissues, anti-inflammatory treatments can be used, such as intra-articular infiltrations of corticosteroids or hyaluronic acid, whose efficacy is also inconstant.

In the medium or long term, the repetition of painful episodes may be at the origin of a phenomenon of sensitization to pain, which develops in the central nervous system. In this case, pain extends beyond the joint and acquires neuropathic features (Lluch Girbés et al., 2016). Central sensitization is also accompanied by exacerbated fatigue, alteration of sleep quality, and mood disorders (Murphy et al., 2011). At this stage, the pain syndrome can become resistant to medical treatment or surgical intervention (Skou et al., 2016) and then other therapeutic approaches must be proposed.

Among these, repetitive transcranial magnetic stimulation (rTMS) of the cerebral cortex has shown interest to treat chronic pain syndrome associated with a central sensitization phenomenon, like fibromyalgia (Passard et al., 2007; Mhalla et al., 2011; Hou et al., 2016; Knijnik et al., 2016). The usual cortical target is the motor cortex (precentral gyrus), and rTMS aims at activating various neural circuits present in this cortical region, which are involved in the sensory, attentional, or emotional control of pain. In clinical practice, rTMS can be an effective therapy in a number of chronic pain syndromes (Lefaucheur, 2016), but has never been used in osteoarthritis-associated pain to our knowledge. In this article, we present the case of a woman suffering from chronic pain related to knee osteoarthritis and resistant to medical treatment. She was not considered eligible for total knee arthroplasty (TKA) surgery and therefore an attempt of rTMS therapeutic trial was proposed. This treatment resulted in a long-lasting analgesic and functional improvement, consistent with a clear relief of the central sensitization phenomenon.

## Case Report

A 71-year-old woman presented bilateral gonalgia predominant on the left side and related to knee osteoarthritis for 20 years. There was a context of overweight (weight: 125 kg; height: 1.60 m; body mass index: 48.8 kg/m^2^). Initially, pain had “mechanical” features, occurring only while standing or walking and limiting physical activities. The diagnosis of bilateral, tri-compartmental knee osteoarthritis predominant on the left side was confirmed radiologically. However, about 4 years ago (2014), knee pain became more intense, diffuse, and permanent, even at rest. In parallel, the patient developed sleep and mood disorders. Intra-articular injections of corticosteroids and hyaluronic acid had only very modest and transient effects. Then, an attempt to use opioids was undertaken for a period of time (fentanyl transdermal patch 25–50 μg/h/day), associated with paracetamol on demand (1,000–3,000 mg/day). This treatment was also ineffective and the patient had to reduce her physical activity even more. In September 2015, her walking distance was limited to 50 m with the aid of a cane. However, X-ray examination did not reveal any aggravation of osteoarthritic lesions.

**FIGURE 1:**
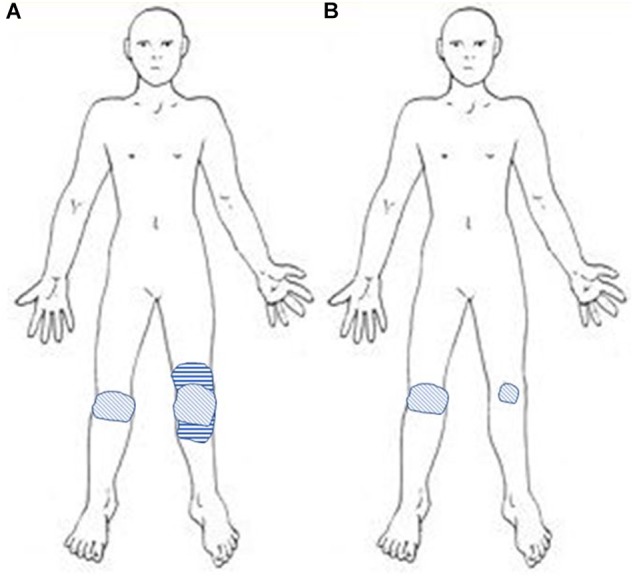
**(A)** Before rTMS: drawing of the extent of mechanical pain (hatched area), surrounded by a region of exacerbated permanent pain with mechanical allodynia evoking central sensitization on the left side only (horizontal stripes). **(B)** After 10 rTMS sessions: the area of mechanical pain is reduced and the zone likely related to central sensitization disappeared on the left side, whereas pain remained unchanged on the right side.

In June 2016, the patient was referred to our center. She was unable to walk and essentially restricted to wheelchair. She scored her average daily pain intensity at 9/10 on a numeric rating scale (NRS), while NRS scores for sleep disorders and fatigue were 7/10 and 6/10, respectively. Anxiety and depression scores were both 12/21 on the hospital anxiety and depression scale (HAD) (Zigmond and Snaith, 1983). The lequesne index of severity for osteoarthritis (LISO) score (Lequesne, 1997) was 20/24, corresponding to an extremely severe handicap. The LISO sub-scores were 7/8 for “pain or discomfort,” 7/8 for “maximum walking distance,” and 6/8 for “activities of daily living.” A rTMS therapeutic trial was proposed, first because total knee replacement surgery was considered too risky for the patient and then because of the clinical arguments in favor of a central sensitization phenomenon. Actually, pain was no more strictly related to joint mobilization, but rather permanent, even present at rest. In addition, pain showed an expanded distribution outside the primarily affected knee joint on the left, as evidenced on pain drawings (Figure 1). This is known to be a reliable way for identifying the occurrence of a central sensitization phenomenon in the context of knee osteoarthritis (Lluch Girbés et al., 2016). There were also burning and tingling sensations, as well as mechanical allodynia and hyperalgesia (but no wind-up phenomenon) in the area of referred pain on the left side. Pain had neuropathic characteristics, as evidenced by a score of 6/10 for the DN4 questionnaire (Bouhassira et al., 2005). A more ambiguous result was observed for the modified painDETECT questionnaire (mPDQ) (Freynhagen et al., 2006), with a score of 13/38, which is the gray zone between improbable neuropathic pain (score < 12) and highly probable neuropathic pain (score > 19). Nevertheless, this mPDQ score was compatible with a central sensitization phenomenon, as the cut-off score was set at > 12 in a recent study of patients with knee osteoarthritis (Hochman et al., 2013). This was further confirmed on the central sensitization inventory (CSI) (Mayer et al., 2012), which assesses both somatic and emotional complaints related to central sensitization and was validated in the context of knee osteoarthritis (Gervais-Hupé et al., 2018). On a short version of the CSI (Nishigami et al., 2018), the patient had an initial score of 20/36 corresponding to moderate/severe central sensitization. In addition, sleep and mood disorders were in the foreground of the clinical picture and no clear worsening of arthritic lesions was observed on X-ray examination. Conversely, on the right side, pain had lower intensity (≤4/10), with only “mechanical” features limited to knee joint.

A rTMS protocol was applied as for the treatment of focal neuropathic pain (Lefaucheur, 2016). The stimulation was delivered over the motor cortex contralateral to the predominant painful region, i.e., right motor cortex stimulation to treat left knee pain. The stimulation parameters for one session were as follows: 20 trains of 70 rTMS pulses delivered at high frequency of 10 Hz (train duration: 7 s; inter-train duration: 55 s), i.e., 1,400 pulses for a session lasting about 20 min. The stimulation intensity was set at 80% of the resting motor threshold, which was determined in a conventional way by means of motor evoked potential recording (Rossini et al., 2015). One rTMS session was performed each month. Assessment was performed during the week after each session (pain, sleep, fatigue) and after the 10th session for all variables, including DN4, mPDQ, CSI, HAD, and LISO.

**FIGURE 2:**
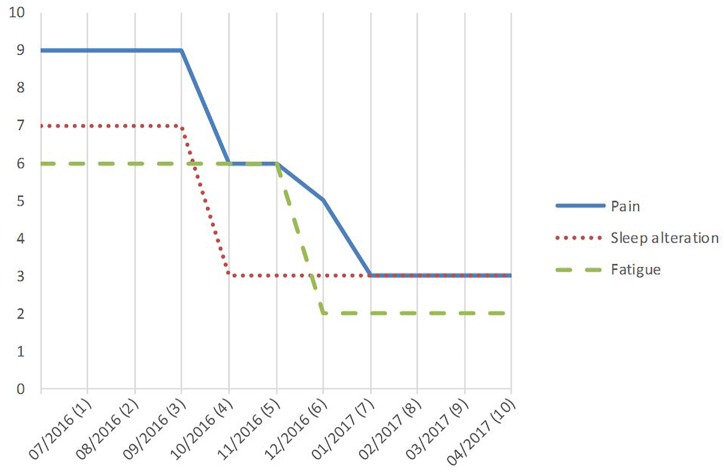
Evolution of average intensity of pain, sleep alteration, and fatigue on a 0–10 numeric rating scale (NRS), between before and the week after 10 monthly sessions (1–10) of repetitive transcranial magnetic stimulation (rTMS) delivered to the right motor cortex.

Pain intensity began to decrease in the week following the third rTMS session, but more clearly after the sixth rTMS session (from 9/10 before to 3/10, 67% improvement) (Figure 2). After 10 sessions, pain was no longer permanent and only occurred when the patient was rising from a sitting position and for walking distance longer than 200 m. Burning sensations, as well as mechanical allodynia and hyperalgesia, disappeared at the left knee. Neuropathic pain scores decreased by 67% (DN4, from 6 to 2/10) to 85% (mPDQ, from 13 to 2/38), and the CSI score by 70% (from 20 to 6/36). Thus, at this stage, pain could no longer be considered neuropathic and central sensitization was absent or at most subclinical (Nishigami et al., 2018). Conversely, pain intensity did not change on the right side.

After the 10th session compared to baseline, sleep disorders (NRS score) also improved by 57% (from 7 to 3/10), fatigue (NRS score) by 67% (from 6 to 2/10), anxiety (HAD score) by 50% (from 12 to 6/21), depression (HAD score) by 42% (from 12 to 7/21), and handicap (LISO score) by 40% (from 20 to 12/24). The LISO sub-scores improved by 71% for “pain or discomfort” (from 7 to 2/8), 14% for “maximum walking distance” (from 7 to 6/8), and 33% for “activities of daily living” (from 6 to 4/8). Analgesic medications were stopped gradually from 3 to 4 months after rTMS therapy initiation to be completely withdrawn at 7 months (after the seventh rTMS session). At this time, body mass index was 48.0 kg/m^2^ and returned to its baseline value (48.8 kg/m^2^) at 10 months. Pain remains fully controlled to date by monthly rTMS sessions. A written informed consent was obtained from the patient for the publication of this case report.

## Discussion

Chronic pain syndrome may worsen in the context of knee osteoarthritis even though the joint lesions are not evolving (Bedson and Croft, 2008), because of a maladaptive sensitization of the pain pathways in the central nervous system (Lluch et al., 2014). Such phenomenon of central sensitization is common in patients with osteoarthritis (Suokas et al., 2012; Lluch et al., 2014), although its propensity to develop varies among patients (Lee et al., 2011). In patients with osteoarthritis, chronic inflammatory nociceptive inputs can dynamically maintain altered central processing (Latremoliere and Woolf, 2009). Central changes particularly affect the descending mechanisms of pain control (impaired descending inhibition and/or enhanced descending facilitation) (Gebhart, 2004; Zambreanu et al., 2005; Arendt-Nielsen et al., 2010; Graven-Nielsen et al., 2012). Such a central dysfunction occurs in various chronic pain conditions, like fibromyalgia, bladder pain syndrome, or irritable bowel syndrome. Clinically, the central sensitization phenomenon makes the pain more intense, permanent and diffuse than it should be, with both temporal and spatial summations. It increases the resistance to local treatments and analgesic medications and is negative prognostic factor for the outcome of total knee replacement surgery (Kurien et al., 2018).

Some psychological factors may explain the discordance between the worsening of pain and the absence of radiographic changes at joint level. Actually, central sensitization is characterized by diffuse psychophysical changes, including unusual fatigue, cognitive difficulties, sleep disturbance, depression and catastrophism, associated with neuropathic pain features, including hyperalgesia and allodynia extending outside the affected joint (Murphy et al., 2011; Lluch et al., 2014, 2017). Central sensitization may be difficult to prove in a clinical setting and requires the identification of both psychological factors and neuropathic pain features. Various questionnaires may help, such the CSI and the mPDQ, of which usefulness was demonstrated in patients with chronic knee osteoarthritis (Hochman et al., 2011, 2013; Moreton et al., 2015). More recently, a list of 14 criteria provided by clinical and sensory examination was proposed for making the diagnosis of central pain sensitization associated with knee osteoarthritis (Lluch et al., 2017). Our patient initially presented 10 of these criteria, including: pain intensity > 5/10, disproportion between pain intensity and structural damage, disproportionate pain after physical activity, inconsistent response to clinical tests, enlarged areas of pain outside the knee, diffuse tenderness, tactile allodynia and hypoesthesia, poor results with analgesics, and unsuccessful response to local interventions.

A trial of rTMS therapy was attempted according to the presence of central sensitization. In such a condition, rTMS can produce analgesia by activating brain circuits that are able to control the hyperactivity of sensitized pain networks. This objective was reached in our patient, since rTMS improved the clinical symptoms related to central sensitization more than those related to the mechanical “aspects” of knee osteoarthritis. For example, “pain or discomfort” LISO sub-score improved much more than the “maximum walking distance” LISO sub-score. In addition, both psychological aspects and neuropathic pain features were dramatically reduced, as well as the CSI score.

Non-invasive cortical stimulation was rarely used to treat knee osteoarthritis, and only by means of transcranial direct current stimulation (tDCS). For example, tDCS (over M1 and/or DLPFC) was used to reduce pain and opioid consumption in postsurgical time after TKA (Borckardt et al., 2013, 2017; Khedr et al., 2017). Motor cortex tDCS was also combined with exercise (Chang et al., 2015, 2017) or peripheral electrical stimulation (Luz-Santos et al., 2017) to promote pain control in knee osteoarthritis. The main results, observed on both provoked and ongoing spontaneous pain, have been recently reported by Ahn et al. (2017, 2018). They performed five daily sessions of anodal tDCS of M1 in patients with knee osteoarthritis and obtained significant analgesic effects compared to sham condition, with lasting clinical benefit for 3 weeks beyond the time of stimulation.

However, to our knowledge, our observation is the first reported case of using rTMS to treat knee osteoarthritis-associated pain syndrome in the long term. Interestingly, in this clinical condition, it has been shown by means of TMS techniques that the corticospinal system had a reduced excitability, in correlation with the intensity of pain, disability, and impairment of the descending controls of pain (Tarragó Mda et al., 2016). Therefore, high-frequency rTMS of the motor cortex could have restored intracortical inhibitory controls in association with pain relief in our patient, as it was previously shown in neuropathic pain (Lefaucheur et al., 2006, 2012) and fibromyalgia or myofascial pain syndromes (Mhalla et al., 2011; Dall’Agnol et al., 2014). Corticospinal excitability studies, based on single- and paired-pulse TMS techniques, deserve to be investigated in future trials in this context.

Usual rTMS protocols include a phase of “induction,” consisting of a daily session for five consecutive days, repeated for one to 2 weeks, followed by one session per week for 4 to 6 weeks (Lefaucheur, 2016). In our patient, we performed only monthly rTMS sessions, especially because the patient was difficult to mobilize. She clearly benefited from this therapy from the third month and one might think that she could have responded well before if the intervals between sessions had been shorter. The benefit continued thereafter throughout the year on various clinical aspects, including daily living activities.

Although a placebo effect cannot be ruled out in the absence of sham procedure, various arguments can be raised against this hypothesis: the absence of previous response to medications and invasive treatments (e.g., intra-articular injections), the progressive clinical improvement which settled down as the rTMS sessions were repeated, and the fact that pain relief occurred only on the left side (contralateral to cortical stimulation), leaving the pain of the right knee unchanged.

## Conclusion

In conclusion, a therapeutic use of motor cortex rTMS is particularly appealing to treat pain associated with knee osteoarthritis, especially when a central sensitization phenomenon is diagnosed and when total knee replacement surgery is risky or even contraindicated. Further sham-controlled study on a large series of patients is of course necessary to confirm this hope.

## Author ConTributions

J-PN, VD, JE, ASM, CM, JN, and J-PL contributed substantial to the conception or design of the work, acquisition, analysis, or interpretation of data. J-PN and J-PL drafted the manuscript or revised it critically for important intellectual content.

## Conflict of Interest Statement

The authors declare that the research was conducted in the absence of any commercial or financial relationships that could be construed as a potential conflict of interest.
